# A novel growth function incorporating the effects of reproductive energy allocation

**DOI:** 10.1371/journal.pone.0199346

**Published:** 2018-06-26

**Authors:** Akihiro Manabe, Takashi Yamakawa, Shuhei Ohnishi, Tatsuro Akamine, Yoji Narimatsu, Hiroshige Tanaka, Tetsuichiro Funamoto, Yuji Ueda, Takeo Yamamoto

**Affiliations:** 1 Graduate School of Agricultural and Life Sciences, The University of Tokyo, Tokyo, Japan; 2 School of Marine Science and Technology, Tokai University, Shizuoka, Shizuoka, Japan; 3 National Research Institute of Fisheries Science, Japan Fisheries Research and Education Agency, Yokohama, Kanagawa, Japan; 4 Hachinohe Laboratory, Tohoku National Fisheries Research Institute, Japan Fisheries Research and Education Agency, Hachinohe, Aomori, Japan; 5 National Research Institute of Far Seas Fisheries, Japan Fisheries Research and Education Agency, Shimizu, Shizuoka, Japan; 6 Hokkaido National Fisheries Research Institute, Japan Fisheries Research and Education Agency, Kushiro, Hokkaido, Japan; 7 Japan Sea National Fisheries Research Institute, Japan Fisheries Research and Education Agency, Niigata, Niigata, Japan; 8 Obama Laboratory, Japan Sea National Fisheries Research Institute, Japan Fisheries Research and Education Agency, Obama, Fukui, Japan; Texas A&M University, UNITED STATES

## Abstract

Ontogenetic growth functions provide basic information in biological and ecological studies. Various growth functions classified into the Pütter model have been used historically, regardless of controversies over their appropriateness. Here, we present a novel growth function for fish and aquatic organisms (generalised *q*-VBGF) by considering an allocation schedule of allometrically produced surplus energy between somatic growth and reproduction. The generalised *q*-VBGF can track growth trajectories in different life history strategies from determinate to indeterminate growth by adjusting the value of the ‘growth indeterminacy exponent’ *q*. The timing of maturation and attainable body size can be adjusted by the ‘maturation timing parameter’ *τ* while maintaining a common growth trajectory before maturation. The generalised *q*-VBGF is a comprehensive growth function in which exponentials in the traditional monomolecular, von Bertalanffy, Gompertz, logistic, and Richards functions are replaced with *q*-exponentials defined in the non-extensive Tsallis statistics, and it fits to actual data more adequately than these conventional functions. The relationship between the estimated parameter values *τ* and *rq* forms a unique hyperbola, which provides a new insight into the continuum of life history strategies of organisms.

## Introduction

Various functions have been developed and used to describe the ontogenetic growth of fishes and aquatic organisms. Among them, monomolecular, von Bertalanffy (VBGF), Gompertz, logistic, and Richards growth functions are used ubiquitously owing to their simplicity and convenience [1–6]. Each of these growth functions is a special case Pütter model [7], which assumes that growth rates are proportional to surplus energy production rates given by the difference between the rates of anabolism and catabolism,

$$dw/dt=\lambda w^{m}-\gamma w^{n},$$ (1)

where *w* is body mass, *t* is time or age, *λ* and *γ* are coefficients, and *m* and *n* are allometric exponents. The general solution of Eq 1 can be given as an incomplete beta function [8].

However, these traditional growth functions do not consider the allocation of energy toward reproduction, which is the ultimate influential event in the life history of an organism. Because the total surplus energy is limited, there is a trade-off of energy allocation between somatic growth and reproduction: commencement of reproduction at an earlier age leads to less somatic growth until maturation—and hence, smaller attainable body size—and vice versa. This trade-off also explains associated strategies in the r/K-selection theory [9], in which r-selected organisms exhibit maturation at younger ages, smaller body sizes, and shorter life spans, while K-selected organisms exhibit maturation at older ages, larger body sizes, and longer life spans.

According to the Pütter model, the reason for the gradual decrease in growth rates with age and the eventual approach to the asymptotic body size is attributable to the difference in the value of power exponents (*m < n*) between anabolism and catabolism: as the catabolism rate approaches the anabolism rate in Eq 1 (i.e. *γw*^*n*^→*λw*^*m*^), the body size approaches the asymptotic size. In contrast, we presume that the primary reason for the decrease in growth rates of organisms is the commencement of reproductive energy allocation after maturation, which exceeds the effects of the difference in the anabolic/catabolic power exponents. This hypothesis can be supported by the much larger attainable body size of triploid fishes and bivalves in comparison to diploids [10, 11], which is attributable to the sterility of triploids and the continuous allocation of surplus energy to somatic growth throughout their life. This hypothesis is also confirmed by one of the driving factors of the notable differences in the attainable body sizes between anadromous fishes and their land-locked types with earlier maturation [12]. Thus, explicit consideration of the schedule and magnitude of energy allocation to reproduction is indispensable for constructing an appropriate somatic growth function.

Organisms exhibit two types of growth patterns: determinate growth and indeterminate growth [13]. Determinate growth is a growth pattern selected mainly by terrestrial endotherms such as mammals and birds [14], in which somatic growth does not occur after maturation. Indeterminate growth is a growth pattern selected mainly by ectotherms such as fishes, reptiles, and amphibians [15], in which substantial somatic growth continues even after maturation. The difference between the two growth patterns might have emerged in the evolution process caused by the trade-off in energy allocation between somatic growth and reproduction [16].

While traditional growth functions do not introduce an explicit formulation for energy allocation, some recent studies have reported new growth models that consider reproductive energy allocation [17–23]. Most of these models are given as biphasic growth functions by separating a life history according to the age of maturation and treating pre- and post-maturation growth stages discontinuously [17, 19, 22, 23]. Other models such as the dynamic energy budget model [18], full life cycle bioenergetics model [21], and state-dependent energy allocation model [20] describe life histories continuously but tend to focus on numerical simulations of ontogenetic growth and energetic dynamics. Although these models perform well to describe the life history of organisms, the lack of simple formulations causes these models not to replace the role of traditional growth functions (e.g. von Bertalanffy growth function).

In the present paper, we propose a novel growth function that satisfies the following requirements: (i) the rate of surplus energy production is related allometrically to body size according to the power law between metabolic rate and body mass [24, 25]; (ii) it explicitly considers the allocation of surplus energy to somatic growth and reproduction; (iii) it is a simple explicit function with a minimal number of parameters and is convenient for practical use; (iv) it can describe various types of growth patterns, from determinate to indeterminate types, and adequately fits to actual data; and (v) each parameter represents a role in a life history strategy, and the function can provide a basic framework for theoretical studies.

## Derivation of the new growth function

To derive a new growth function, we consider the rate of surplus energy production of organisms, which is the difference in energetic gain and cost through anabolism/catabolism and is directed to somatic growth and reproduction. Since the specific metabolic rate (metabolic rate per body mass) of organisms can be generally described by a decreasing allometric function of body mass [24, 25], we assume that the rate of surplus energy production per body mass is expressed as follows:

$$\frac{1}{w}\frac{dS}{dt}=kw^{-\frac{1}{r}},$$ (2)

where *w* is body mass, *S* is surplus energy expressed in the same unit of body mass, *t* is time or age, and *r* and *k* are coefficients (*r* > 0, *k* > 0). Although we could also express Eq 2 in other equivalent forms, e.g. *dS/dt* = *αw*^*β*^, we show the present form of Eq 2 to facilitate the development of a simpler explanation for the derivation of the model in the following part. In this formulation, we implicitly assume that the values of power exponents are identical between anabolism and catabolism, unlike the case of the Pütter model in Eq 1.

**Fig 1:**
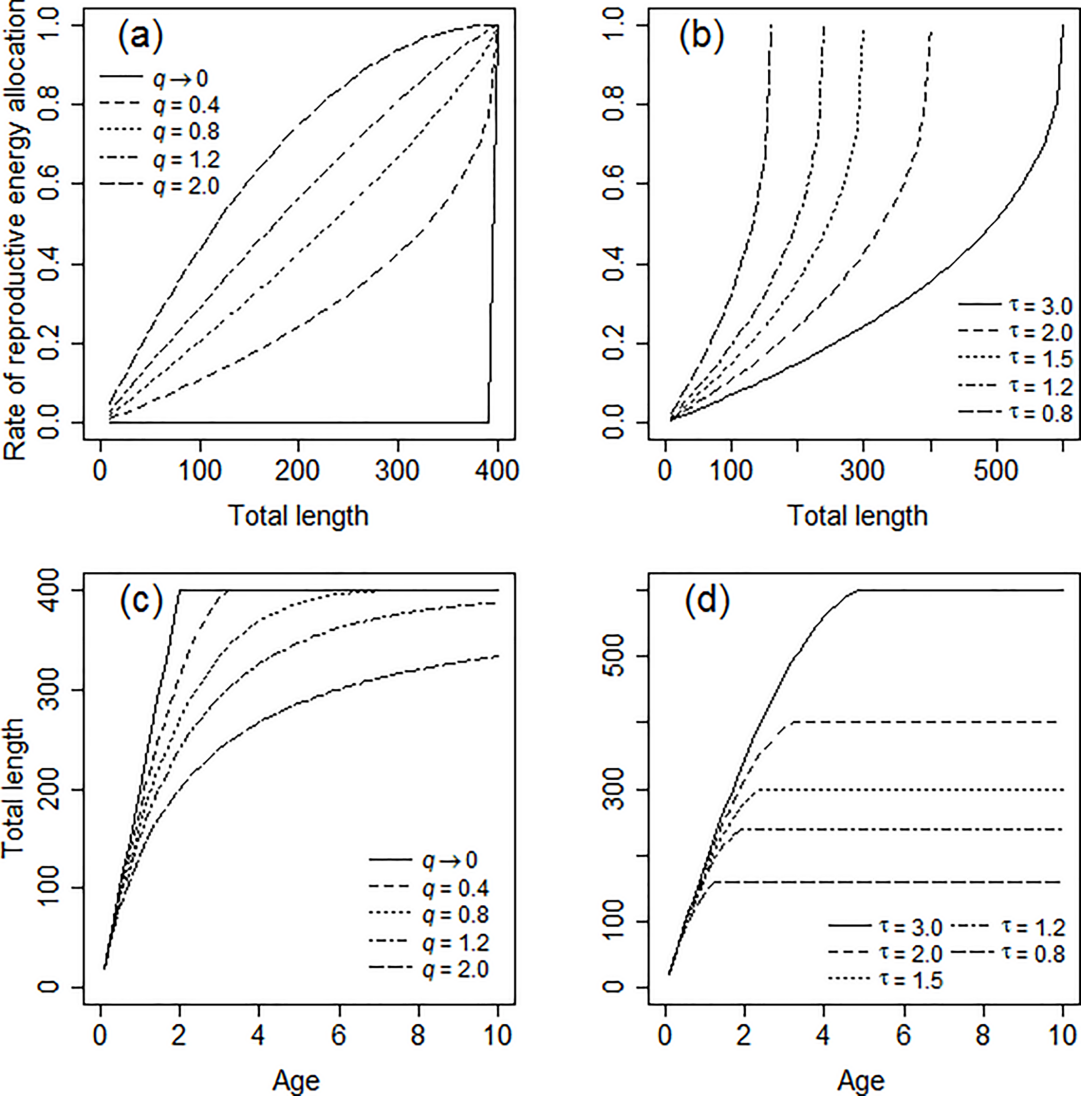
Trajectories of various rates of reproductive energy allocation and somatic growth. Variations in the rates of reproductive energy allocation (a and b) and somatic growth (c and d) with different values of *q* (a and c) and *τ* (b and d) using the present function (Eqs 13 and 14). Values of *q* and *τ* are shown in the graphs, while values for the other parameters are fixed as $\hat{L}$ = 200, *r* = 1, *τ* = 2, and *t*_0_ = 0 for (a) and (b); and $\hat{L}$ = 200, *r* = 1, *q* = 0.4, and *t*_0_ = 0 for (c) and (d).

If the surplus energy is allocated to somatic growth at the rate of a function *g*(*w*), the surplus energy is allocated to reproduction at the rate of 1 –*g*(*w*). The specific rate of somatic growth and the rate of reproductive energy per body mass are expressed as follows:

$$\frac{1}{w}\frac{dw}{dt}=kw^{-\frac{1}{r}}g(w),$$ (3)

$$\frac{1}{w}\frac{dF}{dt}=kw^{-\frac{1}{r}}[1-g(w)],$$ (4)

where *F* is the cumulative energy allocation to reproduction. Although we can assume various functions for *g*(*w*), we put the requirements (iii) and (iv) stated above on *g*(*w*); in fact, many of the differential equations in Eq 3 using candidate *g*(*w*) functions could not be solved or were solved as implicit functions *f*(*w*, *t*) = 0 instead of explicit functions *w* = *f*(*t*), which can cause difficulties in practical applications and future extension. Nevertheless, we could derive a simple explicit function of somatic growth and yet sustain adequate fitting to actual growth and reproductive data if we defined *g*(*w*) by the following power function:

$$g(w)={\left[1-{\left(\frac{w}{w_{\infty }}\right)}^{\frac{1}{r}}\right]}^{q}\;(w\leq w_{\infty },\;q>0,\;r>0),$$ (5)

where *w*_∞_ is the maximum body mass, and *q* is a power exponent. The term (*w*/ *w*_∞_)^1/*r*^ can be considered as a standardised size by adjusting the dimension by the exponent 1/*r*. We can ensure a coherent power form of Eq 3 by setting *g*(*w*) as a power function *g*(*w*) = (1 − *X*)^*q*^ (where *X* = (*w*/*w*_*∞*_)^1/*r*^) and obtain an analytical solution as an explicit function. It is also consistent with the fact that most physiological models (e.g. mathematical formulation of metabolic rate, allometric growth) have been given by power functions. On the basis of this form of *g*(*w*), the trajectory of the rate of energy allocation to reproduction 1 –*g*(*w*) when the value of the power exponent *q* is manipulated is shown in Fig 1A.

By substituting Eq 5 into Eqs 3 and 4:

$$\frac{1}{w}\frac{dw}{dt}=kw^{-\frac{1}{r}}{\left[1-{\left(\frac{w}{w_{\infty }}\right)}^{\frac{1}{r}}\right]}^{q},$$ (6)

$$\frac{1}{w}\frac{dF}{dt}=kw^{-\frac{1}{r}}[1-{\left[1-{\left(\frac{w}{w_{\infty }}\right)}^{\frac{1}{r}}\right]}^{q}].$$ (7)

The formulation of Eq 6 looks somewhat similar to that of the Pütter model because Eq 1 can be transformed as follows:

$$\frac{1}{w}\frac{dw}{dt}=\lambda w^{m-1}\;(1-\frac{\gamma }{\lambda }w^{n-m})\;(0<m<1,\;m<n).$$ (8)

However, Eq 6 is different from Eq 8 in that a new exponent, *q*, is introduced, and the underlying meaning of Eq 6 differs from that of Eq 8, as stated above.

Given that *w* → 0 when *t*→*t*_0_, Eq 6 can be solved as

$$w=w_{\infty }{\left[1-{\left[1-\frac{k}{r{w_{\infty }}^{1/r}}\left(1-q\right)\left(t-t_{0}\right)\right]}^{\frac{1}{1-q}}\right]}^{r},$$ (9)

when 0 < *q* and *q* ≠ 1 (for the derivation of this solution, see Appendix A in S1 Text). To clarify the role of each parameter in the context of life history strategy, we re-parameterised *k* = *rŵ*^*1/r*^ and *w*_∞_ = *ŵτ*^*r*^ (also see Eq 15 to understand the reason for the re-parameterisation: Eq 15 becomes fairly simple by this re-parameterisation) and derived the following novel somatic growth function and the concomitant function for the rate of reproductive energy allocation:

$$w=\left\{ \begin{array}{c} \hat{w}\tau^{r}{\left[1-{\left[1-(1-q)\frac{t-t_{0}}{\tau }\right]}^{\frac{1}{1-q}}\right]}^{r}\;if\;(0<q<1\;and\;t<t_{0}+\frac{\tau }{1-q})\;or\;q>1\\ \hat{w}\tau^{r}\;if\;0<q<1\;and\;t_{0}+\frac{\tau }{1-q}\leq t \end{array}\right.$$ (10)

$$\frac{1}{w}\frac{dF}{dt}=r{\left(\frac{w}{\hat{w}}\right)}^{-\frac{1}{r}}\left[1-{\left[1-\frac{1}{\tau }{\left(\frac{w}{\hat{w}}\right)}^{\frac{1}{r}}\right]}^{q}\right],$$ (11)

The somatic growth function in Eq 10 can be simply expressed as follows:

$$w=\hat{w}\tau^{r}\;{\left[1-{\left[max(0,1-(1-q)\frac{t-t_{0}}{\tau })\right]}^{\frac{1}{1-q}}\right]}^{r},$$ (12)

where max(*x*_1_, *x*_2_) returns the larger value between *x*_1_ and *x*_2_. When *q*→1, Eq 12 becomes the *r*-powered VBGF *w* = *w*_∞_[1 − exp[−*k*(*t*−*t*_0_)]]^*r*^ by the definition of exponential function as $\lim_{n\to 0}{\left(1+nx\right)}^{\frac{1}{n}}=\exp x$.

Because the length–weight relationship is generally described as *w* = *aL*^*b*^ (where *a* and *b* are coefficients [26]), Eqs 11 and 12 can be converted to length-based formulae by replacing *w* and *ŵ* with *aL*^*b*^ and $a{\hat{L}}^{b}$, and defining *r′* = *r*/*b*:

$$L=\hat{L}\tau^{r^{\prime }}\;{\left[1-{\left[max\;(0,1-(1-q)\frac{t-t_{0}}{\tau })\right]}^{\frac{1}{1-q}}\right]}^{r^{\prime }},$$ (13)

$$\frac{1}{aL^{b}}\frac{dF}{dt}=br^{\prime }\;{\left(\frac{L}{\hat{L}}\right)}^{-\frac{1}{r^{\prime }}}\;\left[1-{\left[1-\frac{1}{\tau }{\left(\frac{L}{\hat{L}}\right)}^{\frac{1}{r^{\prime }}}\right]}^{q}\right].$$ (14)

Because the formula of Eq 13 is identical to that of Eq 12, this somatic growth function is closed against allometric transformation, *w* → *aw*^*b*^.

The designated titles of each parameter are listed in Table 1. In this five-parameter growth function in Eq 12 (or Eq 13 for length-based model), the initial growth trajectory before maturation is mainly determined by the growth scale factor *ŵ* (or $\hat{L}$), the growth exponent *r* (or *r′*), and the theoretical age at size zero *t*_0_. This fact can be easily understood if the value of *q* in Eq 12 (or Eq 13) approaches zero, when almost all surplus energy is directed to somatic growth (*g*(*w*) ≈ 1 in Eq 5 if *q* ≈ 0), which corresponds to the situation before maturation. When *q* ≈ 0, Eq 12 is simplified as follows:

$$w\approx \left\{ \begin{array}{c} \hat{w}{\left(t-t_{0}\right)}^{r},\;if\;0<t-t_{0}<\tau \\ \hat{w}\tau^{r}\;(=w_{\infty }),\;if\;\tau \leq t-t_{0} \end{array}\right..$$ (15)

Thus, the growth trajectory is determined only by *ŵ*, *r*, and *t*_0_, and it is free from the effect of *τ* if *t* − *t*_0_ < *τ*. We can also derive a length version of Eq 15 by letting *q* ≈ 0 in Eq 13.

**Table 1:** Designated titles of the parameters.

Parameter	Role and meaning
*ŵ* and $\hat{L}$	Growth scale factors
*r* and *r′*	Growth exponents
*q*	Growth indeterminacy exponent
*Τ*	Maturation timing parameter
*t*_0_	Theoretical age at size zero

In contrast, the maturation timing parameter *τ* in Eqs 12 and 13 adjusts the timing (early/late) of maturation (Fig 1B and 1D), and the growth indeterminacy exponent *q* modulates the trajectory of energy allocation to reproduction (Fig 1A). We can describe a wide range of post-maturation growth trajectories from determinate to indeterminate growth in a continuous manner by adjusting the value of *q* (Fig 1C). Although Eqs 12 and 13 theoretically approach or reach the maximum body mass *w*_*∞*_ (= *ŵτ*^*r*^) and length *L*_*∞*_ (= $\hat{L}\tau^{r}$) when *t* → *∞*, respectively, the indeterminate growth pattern can be described in practical ranges of *t* << *∞* when *q* >> 0, if the values of *w*_*∞*_ (= *ŵτ*^*r*^) and *L*_*∞*_ (= $\hat{L}\tau^{r}$) are sufficiently large (Fig 1C). The ‘maximum body sizes’ *w*_*∞*_ and *L*_*∞*_ can be either maximum attainable body sizes or theoretical asymptotic body sizes according to the values by the ‘max’ function in Eqs 12 and 13. When the max function returns zero, *w*_*∞*_ and *L*_*∞*_ are the maximum attainable body sizes. In contrast, when the max function returns positive values even when *t* approaches ∞, *w*_*∞*_ and *L*_*∞*_ are the theoretical asymptotic body sizes.

By using the *q*-exponential (exp_*q*_) defined in the non-extensive Tsallis statistics [27, 28],

$$\exp_{q}x≔{\left[1+(1-q)x\right]}^{\frac{1}{1-q}},$$ (16)

where lim_*q*→1_ exp_*q*_*x* = exp*x* by the definition of exponential function, we can extend the exponential family into a family of power functions (*q*-exponential family) [28–30]. The present growth formula in Eq 12 can be rewritten as follows:

$$w=w_{\infty }{exp}_{1-\frac{1}{r}}\left[-r\;{exp}_{q}(-\frac{t-t_{0}}{\tau })\right].$$ (17)

This form is considered as an extended formula in which two exponentials in the Gompertz formula

$$w=w_{\infty }exp\left[-exp[-K(t-t_{0})]\right]$$ (18)

or an exponential in the Richards formula

$$w=w_{\infty }\;{\left[1+pexp[-K(t-t_{0})]\right]}^{-\frac{1}{p}}$$ (19)

are replaced by *q*-exponentials and re-parameterised (for the derivation, see Appendix B in S1 Text). Likewise, VBGF (including the monomolecular function) and its cubic and logistic growth formulae can also be extended to Eq 17 by using the *q*-exponential, because these traditional growth formulae are special cases of the Richards formula: i.e. Eq 19 is identical to VBGF, its cubic, Gompertz, and logistic formulae when *p* = −1, −1/3, 0, and 1, respectively [5]. Thus, the growth formula in the present paper can be considered a comprehensive formula that includes all these traditional growth formulae. Given that VBGF is currently the most widely used growth formula, and Eq 12 can be directly derived by taking a *q*-exponential of the *r*-powered VBGF *w* = *w*_∞_[1 − exp[−*k*(*t*−*t*_0_)]]^*r*^ instead of the traditional cubic form, we designate this function as ‘generalised *q*-VBGF’.

The generalised *q*-VBGF can be extended to several forms. Various extended forms of the model, such as those with seasonal growth variation and/or constraints to pass through certain points (e.g. size and age at hatching, maturity, and any other given stages), are derived in the Supplementary Discussion in S1 Text.

## Materials and methods

### Applied data

To test the performance of the generalised *q*-VBGF, we applied Eqs 13 and 14 to length (and gonad weight) at age data from two fish (the willowy flounder [*Tanakius kitaharae*] and the Alaska pollock [*Gadus chalcogrammus*]), one mammal (the Antarctic minke whale [*Balaenoptera bonaerensis*]), and one crustacean (the snow crab [*Chionoecetes opilio*]) species (for details and origin of the data, see Supplementary Material in S1 Text).

For fish and mammal species, we estimated the average growth trajectories across individuals. For crustacean species, we estimated the growth trajectories using the average body size at instar (age counted by number of moults) of each phenotypic group with different terminal instars at which sexual maturity is attained, no further moulting occurs, and multiple reproduction events occur.

### Application methods

To evaluate the performance of the generalised *q*-VBGF, we applied three methods to fit growth functions to the data. The simple ‘standard fit’ method using the generalised *q*-VBGF (Eq 13) and other traditional growth functions was used to estimate male and female somatic growth independently. To compare the result of fitting, four traditional functions (VBGF, logistic, Gompertz, and Richards functions) and the extended-VBGF [22], which was derived considering energy allocation to reproduction, were also fitted to the data. Additional data of larval size at age for the willowy flounder and the Alaska pollock sourced from papers by Fujita [31] and Yoklavich & Bailey [32], respectively, were supplemented to validate the performance of the growth functions to cover the entire life cycle from hatching to old age.

The ‘shared parameter fit’ using the generalised *q*-VBGF (Eq 13) was applied to estimate the somatic growth of males and females (or different cohorts of the minke whale or different phenotypic groups of the snow crab with different terminal instars) dependently, with a few shared parameters ($\hat{L}$, *r*, and *t*_0_), to elucidate the common growth trajectory before maturation. Other parameters (*q*, *τ*) were estimated for each sex (or cohort or phenotypic group).

The ‘simultaneous fit’ by the generalised *q*-VBGF applied somatic growth and reproductive energy allocation functions (Eqs 13 and 14) to growth data and gonad weight data simultaneously. For this fit, we gave no individual correspondence of body-size data to gonad-weight data: they were simultaneously fitted as group data. Because the energy cost to produce a unit of somatic tissue substantially differs from that for a unit of gonadal tissue [33], we introduced a conversion factor, *c*, into the left-hand side of Eq 14 and fitted the expected value of *c*∙*dF/dt* calculated from Eq 14 to the gonad weight data.

The fitting was performed by a maximum likelihood method with normal distribution using the R (CRAN project R 3.4.4) package ‘stats4’. For the ‘standard fit’ and ‘shared parameter fit’ of the Alaska pollock and willowy flounder, we defined the standard deviations of growth data as a function of age (*σ* = *a* + *bt*, where *t* is age, and *a* and *b* are coefficients) to match the heteroscedasticity of the data. For the snow crab, standard deviation was estimated as a single parameter, since the data used were average size at age (instar). For the Antarctic Minke whale, the standard deviation at each age was given from the original data. For the ‘simultaneous fit’ of the Alaska pollock, a maximum likelihood method with lognormal distribution was used, considering the distribution of both gonad and growth data. To compare the performance of the models, the Akaike information criterion (AIC [34]) and Bayesian information criterion (BIC [35]) were used. For the convenience of the readers, an MS-Excel worksheet for application of the generalised *q*-VBGF, as well as its extended forms, is provided in S1 File.

To investigate the characteristics of parameters to depict different life history strategies, the relationship between the estimated parameters *τ* and *rq*, as well as between *τ* and *q*, was examined. While the growth indeterminacy exponent *q* mainly governs the curvature of the growth trajectory after maturation, the growth exponent *r* affects the overall curvature of the trajectory throughout the life history, as *r* is an overall exponent of the bracket in the right-hand side of Eqs 12 and 13. Therefore, we may expect that *rq* rather than *q* can capture the degree of curvature of the growth curve after maturation more appropriately, and it is a better choice to be employed as an axis to discuss life history strategies of various species across taxa. The appropriateness of *rq* as an index of indeterminate growth can be supported by considering the logarithmic ratio Φ of size at maturity (when *t* = *τ + t*_0_) against the theoretical maximum size (when *t* → *∞*);

$$\Phi =ln\frac{w_{\infty }}{w(\tau +t_{0})}=ln\frac{\hat{w}\tau^{r}}{\hat{w}\tau^{r}{\left(1-q^{\frac{1}{1-q}}\right)}^{r}}=rln\frac{1}{1-q^{\frac{1}{1-q}}}.$$ (20)

By setting $Q=ln\frac{1}{1-q^{\frac{1}{1-q}}}$, then Φ = *rQ*. Considering *Q* to be a monotonic increasing function of *q* (S1 Fig), the size at maturation is small relative to the theoretical maximum size when *rq* is high, which indicates indeterminate growth. When *rq* is low, the size at maturation is close to the theoretical maximum size, which indicates determinate growth.

## Results

**Fig 2:**
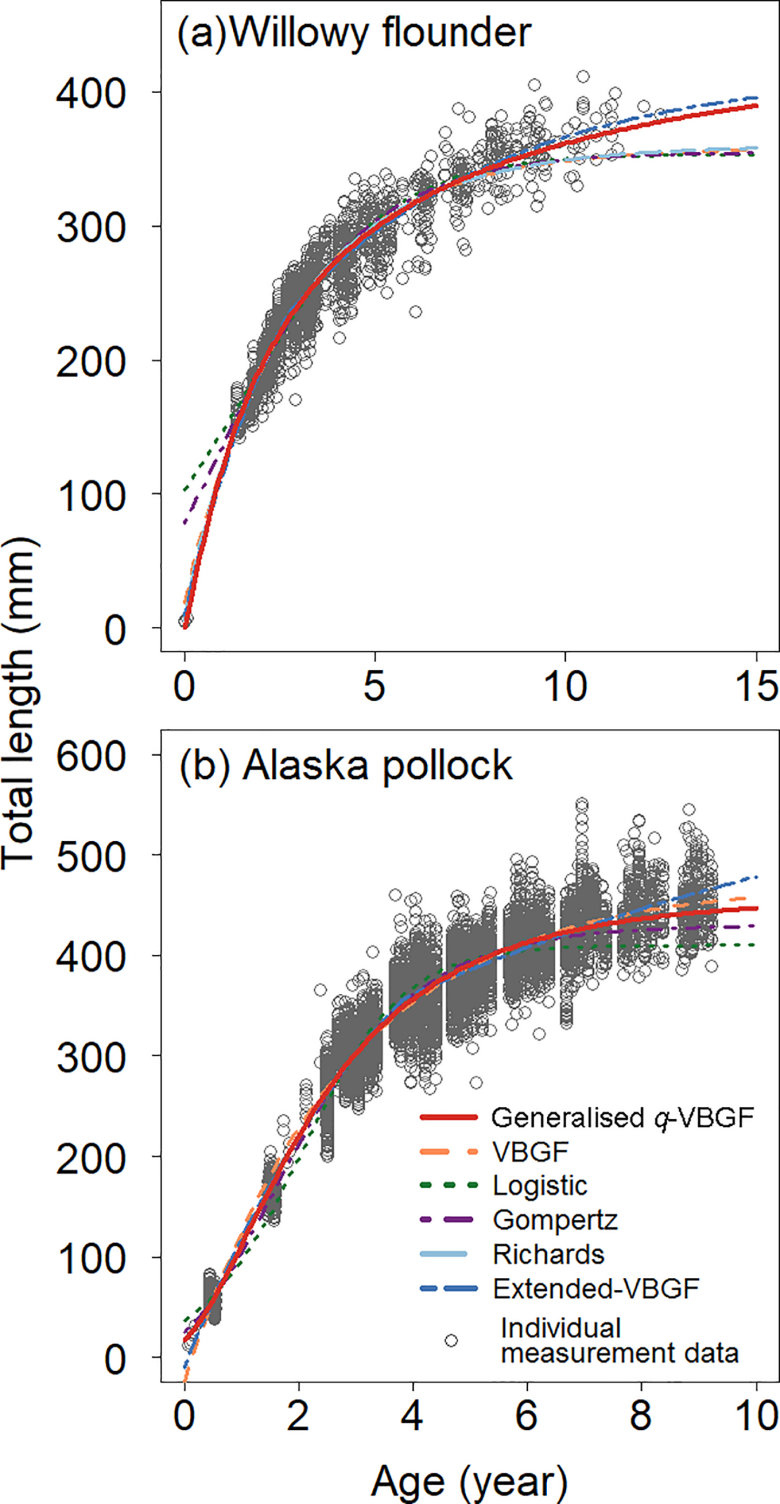
Estimated growth curves using the ‘standard fit’. (a) Female willowy flounder (*Tanakius kitaharae*) and (b) female Alaska pollock (*Gadus chalcogrammus*) are shown.

In the simple ‘standard fits’, Eq 13 and other traditional growth functions are fitted to the data from the willowy flounder and the Alaska pollock (Fig 2). The growth trajectories representing fitted versions of the generalised *q*-VBGF and the extended-VBGF tracked the data plots well (for estimated parameter values and AIC/BIC, see S1 Table). The trajectories showed distinct indeterminate growth patterns: substantial growth was sustained even after maturation. As the optimal functions, AIC and BIC selected the generalised *q*-VBGF in two cases (male willowy flounder and female Alaska pollock), the extended-VBGF in one case (female willowy flounder), and the Richards function in one case (male Alaska Pollock), although the small difference in BIC for the male Alaska pollock between the Richards function and the generalised *q*-VBGF suggested that the generalised *q*-VBGF was equivalently appropriate. None of the traditional functions that did not consider energy allocation performed well, with the exception of the one case (male Alaska pollock) of the Richards function. It seems that most of the traditional models tended to fit to the data-rich portion well in the middle-age growth trajectory; however, they failed to track data-sparse portions in early stages and underestimated the size of older individuals that exhibit indeterminate growth.

**Fig 3:**
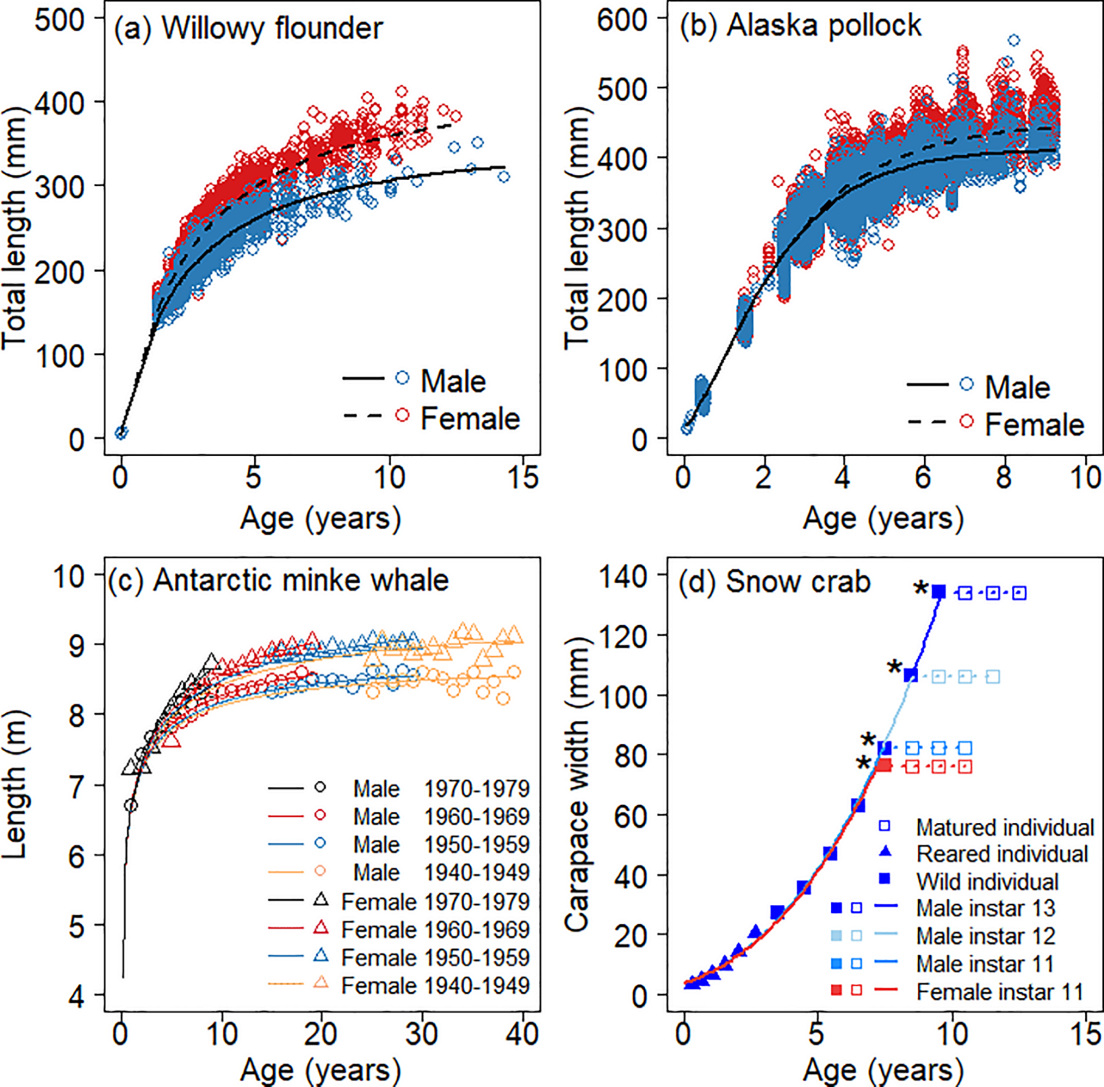
Estimated growth curves using ‘shared parameter fits’. Generalised *q*-VBGF are fitted using ‘shared parameter fits’ between males and females of (a) the willowy flounder (*Tanakius kitaharae*) and (b) the Alaska pollock (*Gadus chalcogrammus*), among males and females of four decadal cohorts of (c) the Antarctic minke whale (*Balaenoptera bonaerensis*), and among male and female groups with different terminal instars for (d) the snow crab (*Chionoecetes opilio*) using the generalised *q*-VBGF. For the snow crab, the solid squares represent the average size at each instar, and the solid squares with a black asterisk on the left side represent the average size at the terminal instar for each group. The blank squares represent the size at age after terminal moulting, and the dotted line represents the fitted growth curve in the period with repeated reproductions, during which no further moulting or somatic growth occurs.

Next, we conducted ‘shared parameter fits’ to the data from the willowy flounder and the Alaska pollock (Fig 3A and 3B, S1 Table). The differences in the growth indeterminacy exponent *q* and the maturation timing parameter *τ* described male and female post-maturation growth trajectories differently, although the growth in immature stages was shared between both sexes. The higher values of *τ* in female willowy flounder and Alaska pollock than those of the respective males exhibited growth trajectories with longer initial growth periods, later maturity timing, and greater length after maturation in females than in males. No conventional growth functions without the consideration of reproductive energy allocation enabled such a usage as the ‘shared parameter fits’ because changes in the parameter values related to the growth trajectory after maturation also affect the growth trajectory before maturation, and vice versa, in the conventional functions.

In contrast to teleosts, the results of the ‘shared parameter fits’ in the data from the Antarctic minke whale and snow crab showed determinate growth patterns (Fig 3C and 3D, S2 and S3 Tables). Here, in addition to $\hat{L}$, *r*, and *t*_0_, parameter *q* was also shared in each species, except in female snow crabs. Differences in only the maturation timing parameter *τ* in the generalised *q*-VBGF could represent the differences in growth trajectories well among male snow crabs with different terminal instars and in different decadal cohorts of minke whales. The notably small values of *q* (S3 Table) in the snow crab implied that, as shown in the case of *q* ≈ 0 in Fig 1A and 1C, the species switched to allocate almost all surplus energy to reproduction and ceased somatic growth (graphed horizontally in Fig 3D) after their terminal moulting, which is associated with sexual maturation. This also indicated that the growth trajectory of the snow crab before maturation was adequately represented by the simple allometric growth function in the length-converted version of Eq 15, which meant that the assumption of Eq 2 was appropriate: the rate of surplus energy production was allometrically related to body mass, and the values of power exponents on anabolism and catabolism are not quite different. No other growth functions, except the generalised *q*-VBGF, could describe such a completely determinate growth trajectory.

**Fig 4:**
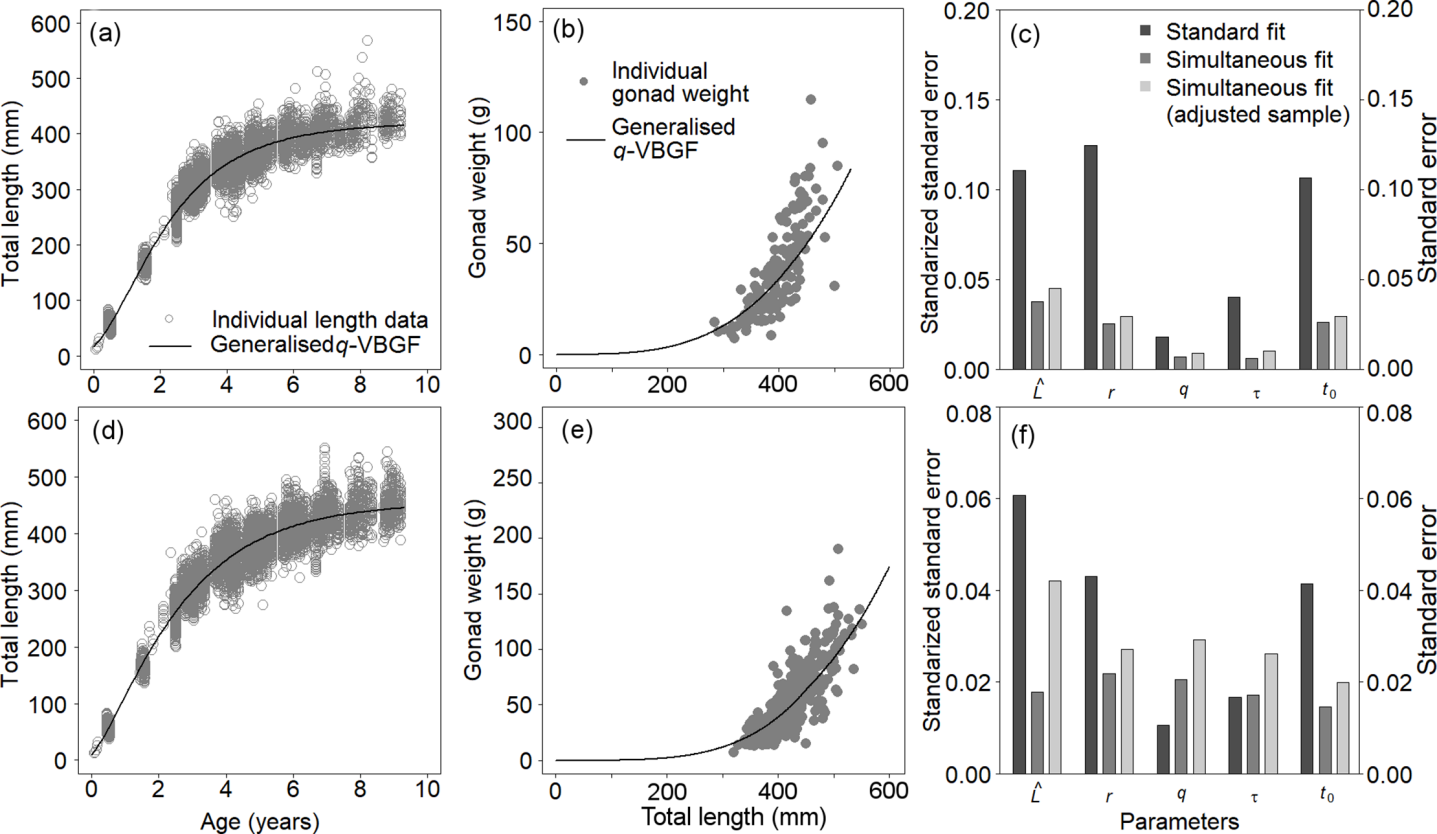
Estimated growth and reproductive curves using the ‘simultaneous fit’. Shown are the estimation of somatic growth and reproductive allocation in gonad weight using ‘simultaneous fit’ for male (a and b) and female (d and e) Alaska pollock (*Gadus chalcogrammus*). The standard errors are compared for each parameter among the ‘standard fit’, ‘simultaneous fit’, and ‘simultaneous fit’ with adjusted number of samples by the generalised *q*-VBGF for (c) male and (f) female Alaska pollock. The standard errors of *t*_0_ show the actual values and the standard errors of *ŵ*, *r*, *q*, and *τ* show the actual values divided by the estimated parameter values for standardization.

The result of the ‘simultaneous fit’ showed appropriate fitting to both the somatic growth and gonad weight data of the Alaska pollock (Fig 4A and 4B, S4 Table). Most of the standard errors of the estimated parameters by the ‘simultaneous fit’ were much smaller than those by the ‘standard fit’ (Fig 4C and 4F, S1 and S4 Tables). This result might have been caused by a simple increase in the sample size. However, a reduction in the sample size to match that of the ‘standard fit’ by removing growth data randomly still showed much smaller standard errors (Fig 4C and 4F, S4 Table). This result suggested that the ‘simultaneous fit’ to somatic growth and gonad weight data could improve the precision of the estimated growth curves by including information from multiple sources (somatic growth and gonad data, size at maturation, size at hatch, etc.).

**Fig 5:**
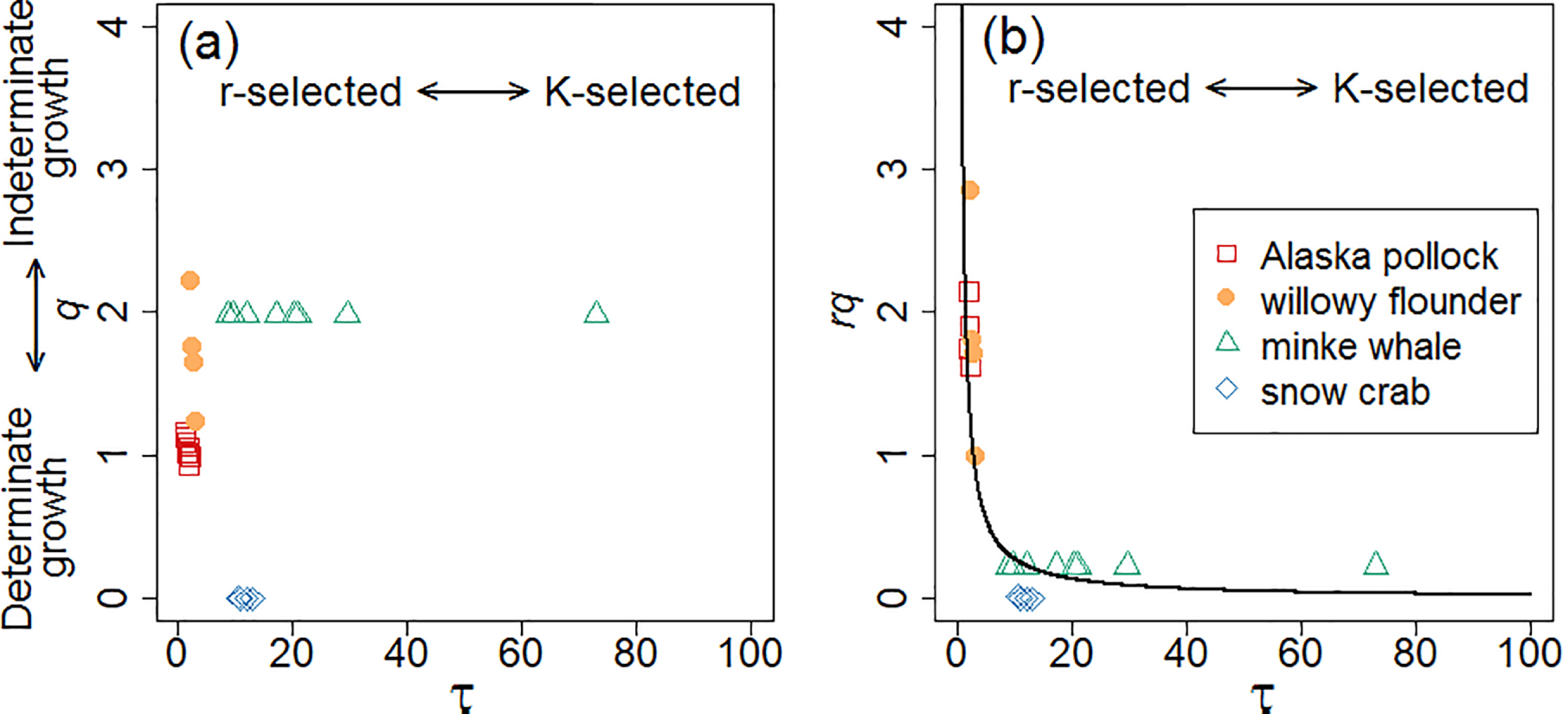
Relationship between estimated parameter values. (a) The maturation timing parameter *τ* and growth indeterminacy exponent *q*, and (b) *τ* and q*r (*q* multiplied by growth exponent *r*) are shown. The solid curve in (b) is a hyperbola fitted to the plots. Arrows indicate axes for the r/K selection theory (horizontal) and determinate–indeterminate growth continuum (vertical).

Fig 5 shows the relationship between the estimated values of *τ* and *q*, as well as *τ* and *rq* (Fig 5). When *q* was multiplied by *r*, the relationship collapsed into a narrow range around a hyperbola (Fig 5B). The value of *rq* for the minke whale also collapsed into the hyperbola in contrast to the value of *q*, reflecting the small value of *r* for the minke whale (S2 Table) compared to the others. Thus, parameter values estimated by the generalised *q*-VBGF can be utilised to quantitatively address life history strategies in a coordinate drawn by the combination of the two axes, i.e. timing of maturation (*τ*) and degree of determinate/indeterminate growth (*rq*).

## Discussion

In the present paper, we have developed the generalised *q*-VBGF, which could be utilised as a standard growth function. The generalised *q*-VBGF satisfies the requirements presented above by (i–iii) providing a pair of explicit growth functions: Eqs (12 and 11) and (13 and 14); (iv) showing that the model could be appropriately fitted to the actual growth data of both indeterminate and determinate growth species; and (v) representing a quantitative role of the parameters to describe life history strategies.

**Fig 6:**
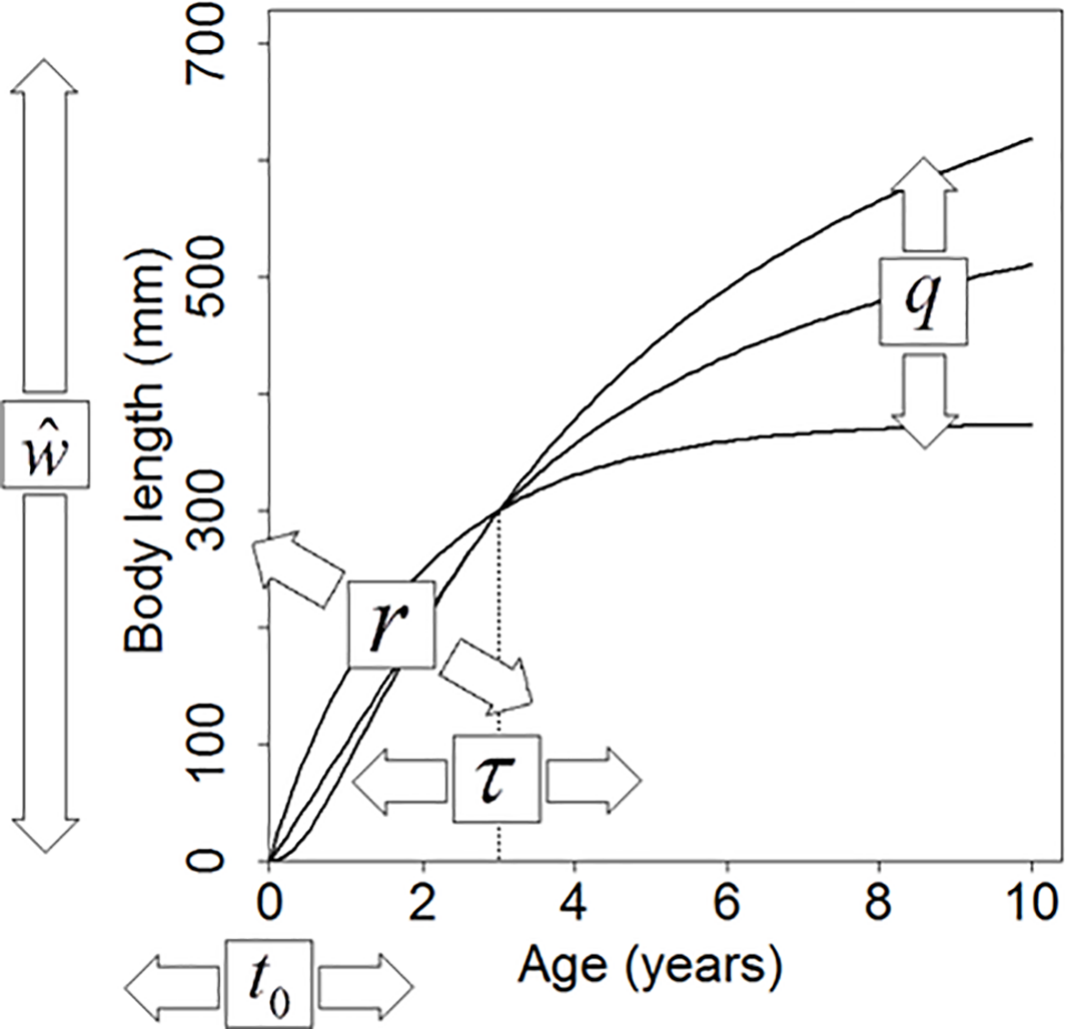
Role of five parameters to depict growth curves, in which growth rates change between pre- and post-maturation. As an example of the five parameters, those of the generalised *q*-VBGF are shown. The pair of arrows indicates that each parameter in the box can adjust the growth trajectory in the pointed direction.

The generalised *q*-VBGF uses five parameters instead of the traditional three- or four-parameter models. While the third goal of our study aims at formulating a simple and explicit function, we surmise that five is the minimal number of parameters to flexibly describe the growth of organisms with changing growth rate between pre- and post-maturation (Fig 6). When the growth rate differs between pre- and post-maturation, the age at maturation is the critical point; hence, one parameter should be dedicated (*τ*). To depict pre-maturation growth, one additional parameter is set to express the theoretical age when *w* = 0 (*t*_0_), and another parameter is required to determine whether the trajectory takes a straight, convex, or concave shape (*r*). To describe post-maturation growth, one parameter is required to determine the shape of the post-maturation growth trajectory (*q*). Finally, another parameter (*ŵ* or *w*_*∞*_) is required to set the overall size scale. Thus, the nature of possible life histories should be incorporated in growth models with at least five parameters with respect to the degree of freedom. In contrast, when the traditional three- or four-parameter models are fitted to follow the pre-maturation data, the trajectory does not fit well to the post-maturation data. In addition, when the data concentrate at the post-maturation stage, the trajectory mainly follows post-maturation growth and will not estimate the pre-maturation growth appropriately (Fig 2). Without the consideration of reproductive investment, these traditional models are only useful as limited phenomenological descriptions [36].

The growth curve obtained by the generalised *q*-VBGF showed appropriate fitting to all the examples that we provided. The ability of the model is also supported by the accurate fitting of the actual data of the snow crab with shared pre-maturation growth, which cannot be obtained by the traditional models, as well as the simultaneous fitting that significantly narrowed the standard errors of the estimated parameters. Although other models showed satisfactory results in a few cases; only the five-parameter models, the generalised *q*-VBGF, and the extended-VBGF performed well in overall datasets, with slight differences among cases (Figs 2 and 3, S1 Table). The generalised *q*-VBGF described a continuous growth trajectory with a smooth change in growth rate at maturation to show the mean growth rate of a population. Considering the growth variation among individuals within a population, acute change in growth rate might be inappropriate to describe the mean growth rate of a population. In contrast, the extended-VBGF showed a discontinuous trajectory, which is appropriate to describe individual growth, because switching (on/off) of energy allocation by an individual leads to a sudden change in the growth rate. However, the extended-VBGF is impractical because reproductive energy by the extended-VBGF gradually declines in most of the post-maturation stages, as shown in the simulation provided by the original study [22]. While natural reproductive senescence—and hence, rapid decline in reproductive investment—can occur at the terminal stage of longevity, reproductive energy and fecundity typically increases with age and body size throughout the middle stage of life history in iteroparous fishes [37].

When the size-at-age data of individuals at the initial stage and/or old age are deficient in practical applications, the precision of estimated parameters will not be high, especially when the number of parameters is large with respect to the low quality of data. In such situations, it may be helpful to set some constraints for the growth curve to pass through certain points (e.g. size at birth, size at maturity, maximum size) using the forms shown in the Supplementary Discussion in S1 Text, and/or fixing the values of parameters *r* and/or *q* to values derived from taxonomically related species or reliable sources. When the gonad data are available with size at age data, applying the ‘simultaneous fit’ can significantly increase the precision, as shown in Fig 4C and 4F.

The generalised *q*-VBGF can describe typical classifications of life history strategies by adjusting the values of the maturation timing parameter *τ* and/or the growth indeterminacy exponent *q* (or *rq*) (Fig 1). With respect to the r*/*K-selection theory [9], growth trajectories in r-selected (early maturation) and K-selected (late maturation) strategies can be described by low *τ* and high *τ*, respectively, as shown in the cases in Fig 1D. In Fig 5 we show the axis of the r/K selection in parallel with the horizontal axis of the maturation timing parameter *τ*.

The generalised *q*-VBGF can describe a growth pattern from determinate to indeterminate growth by changing the value of the growth indeterminacy exponent *q* (Fig 1A and 1C) and the resultant value of *rq*. When *q* → 0, most of the surplus energy during the growth stage before maturation is allocated to somatic growth, while most energy is allocated to reproduction after maturation. Sibly *et al*. [38] defined such a strategy as ‘bang-bang control’ of surplus energy allocation, which originated from the technical term in control engineering: changing the input as either zero or maximum. In contrast, when *q* >> 0 in the generalised *q*-VBGF, a fraction of surplus energy is allocated to reproduction, while somatic growth remains substantial, resulting in the initiation of the first reproduction at an earlier age, with lesser fecundity and successive indeterminate growth with repeated reproductions.

A typical determinate growth pattern depicts ceased somatic growth after maturation, which maximises theoretical lifetime fecundity [39]. Katsukawa *et al*. [40] showed from their theoretical study that determinate growth is selected as the optimal strategy when the environment is relatively stable and constant, whereas indeterminate growth is selected when the environment is stochastic and fluctuates to a greater degree. While determinate growth is employed in mammals and birds whose neonatal survivability is relatively high, indeterminate growth is employed in fishes and marine invertebrates, which spawn many small eggs into the surrounding water freely, to adapt to highly unstable environments with unpredictable future survival [39–42]. The value of the parameter *q* modulates the degree of the growth patterns between these strategies.

We showed a way to quantitatively address different life history strategies by using the parameter values of the generalised *q*-VBGF in a coordinate drawn by the combination of the two axes, i.e. timing of maturation (*τ*) and degree of determinate/indeterminate growth (*rq*). If the hyperbolic relationship between *τ* and *rq* can be supported by using data for various species across taxa, then *τ × rq* becomes constant and can be considered as a new life history invariant [43, 44]. However, the number of species analysed in the present study is too low to discuss the scattering range of plots in Fig 5 and the detailed differences in life history strategies among the plots; therefore, the accumulation of more data based on the application of the generalised *q*-VBGF is required. We expect that the generalised *q*-VBGF will become a useful tool for describing and compiling basic growth and reproductive patterns of various organisms and for revealing the consequences of trade-offs between different life history strategies with respect to lifetime energy allocation.

## Supporting information

S1 Fig Relationship between *q* and *Q*. The figure shows that *Q* is a monotonic increasing function of *q*.
(TIF)

S2 FigAn example of the growth curve depicted by equations A and B in S1 Text.The growth trajectory shows a wavy shape caused by fluctuation in seasonal growth rate. Values of parameters are set as $\hat{L}$ = 150, *r′* = 2.1, *q* = 1.6, *τ* = 0.7, *t*_0_ = −0, α = 1, and *t*_1_ = 1.(TIF)

S1 Table Parameters estimated for the growth functions using the ‘standard fit’ and the ‘shared parameter fit’. Growth functions are fitted to the willowy flounder *Tanakius kitaharae* (*n* = 1905 and 1809 for male and female, respectively) and the Alaska pollock *Gadus chalcogrammus* (*n* = 6062 and 8554 for male and female, respectively). ΔAIC and ΔBIC show the difference of AIC and BIC value between the generalised *q*-VBGF and others, respectively. The value is positive when the generalised *q*-VBGF is more appropriate. Numbers in parenthesis are the standard errors.
(XLSX)

S2 Table Parameters estimated by the ‘shared parameter fit’ of the generalised *q*-VBGF to the Antarctic minke whale *Balaenoptera bonaerensis*. Numbers in parenthesis are the standard errors.
(XLSX)

S3 Table Parameters estimated by the ‘shared parameter fit’ of the generalised *q*-VBGF to the snow crab *Chionoecetes opilio*. Numbers in parenthesis are the standard errors.
(XLSX)

S4 Table Parameters estimated by the ‘simultaneous fit’ of the generalised *q*-VBGF to the Alaska pollock *Gadus chalcogrammus*. To justify the comparison of the confidence interval, size at age data are removed randomly (male = 154, female = 303) to adjust and match the total sample size of the gonad at length data. The number in parenthesis is the standard error.
(XLSX)

S1 Text Supplementary materials, discussions, and appendices A-D.
(DOCX)

S1 File Template for the generalised *q*-VBGF. An MS-Excel worksheet template for the calculation of the generalised *q*-VBGF with three different fitting methods and four extended forms.
(XLSX)
