# Supplementary figures and images for: A novel growth function incorporating the effects of reproductive energy allocation
Source: PLoS One. 2018 Jun 26;13(6):e0199346. doi: 10.1371/journal.pone.0199346 (PMC6019753; doi:10.1371/journal.pone.0199346)

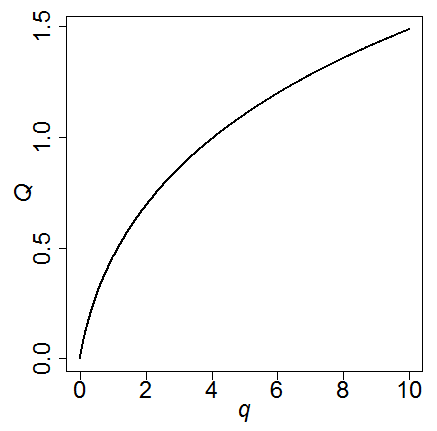

Supplement: S1 Fig — The figure shows that Q is a monotonic increasing function of q. (TIF) [file pone.0199346.s001.tif]
