# Supplementary material for: A novel growth function incorporating the effects of reproductive energy allocation
Source: PLoS One. 2018 Jun 26;13(6):e0199346. doi: 10.1371/journal.pone.0199346 (PMC6019753; doi:10.1371/journal.pone.0199346)
Supplement: S1 Text — Supplementary materials, discussions, and appendices A-D. (DOCX) [file pone.0199346.s007.docx]

**S1 Text: Supporting Information for A novel growth function incorporating the effects of reproductive energy allocation**

The MS Excel worksheet “S1_File.xlsx” is provided as examples and templates for the applications of the generalized *q*-VBGF. This Excel worksheet contains examples for the “standard fit” with the least squares method, “standard fit” with the maximum likelihood method, and “shared parameter fit” as well as “seasonal growth functions” (equations A and B) and three deformed functions of the generalized *q*-VBGF (equations C-E) applied to artificial data.

**Supplementary Material**

The size-at-age data for the willowy flounder (*Tanakius kitaharae*) were obtained from catches by commercial bottom trawl fisheries from the coastal area of Fukushima Prefecture, Japan, from 2002 to 2006. Of the 3,704 individuals evaluated, 1,900 were males and 1,804 were females. To supplement the size at age data for the early stage, five data points were supplemented from Fujita [31].

For the Alaska pollock (*Gadus chalcogrammus*), size at age and gonad weight at age data from a 2005 cohort were used. Samples were collected from catches by offshore bottom trawl fisheries from September to May in successive years between 2006 and 2014 off the Pacific coast of eastern Hokkaido, Japan. Survey data were also used to supplement data for young-of-the-year to two-year-old individuals. Surveys were conducted in June and July from 2005 to 2007 in eastern and southern Hokkaido using a semipelagic/bottom trawl. Of the 12,718 individuals evaluated, 4,852 were males, 7,354 were females, and 512 were unsexed. To supplement the size at age data for the pre-maturation stage, individual data with less than 2.5 years old from the 2004 cohort from the same survey were added. Of the 835 individuals supplemented, 147 were males, 137 were females, and 551 were unsexed. For the early stage, five data points representing the larval stage were supplemented from Yoklavich and Bailey [32]. For the ‘simultaneous fit’, gonad weight data from 154 males and 303 females collected in December during the peak of the reproductive season were used.

The data used for the Antarctic minke whale (*Balaenoptera bonaerensis*) were obtained from the literature [45]. Mean body length at age for each sex in each decadal year class group (1940–1949, 1950–1959, 1960–1969, and 1970–1979) was analysed. The data were collected as a part of the Japanese Antarctic whaling expedition from 1971 to 1983 in offshore Antarctica.

The size-at-age data for the snow crab (*Chionoecetes opilio*) were obtained from laboratory rearing experiments under 1°C for the first to sixth instars, and the accumulated information by routine stock assessments for the seventh to thirteenth instars [46, 47]. Average carapace width and estimated age for each instar for each sex were analysed. A minor gap in the growth trajectories between the reared and wild individuals was observed; however, it conferred little effect on the entire estimation of the growth curve.

**Supplementary Discussion**

The generalized *q*-VBGF can be extended to several forms. If there is a seasonal variation in growth rates, we can introduce a cumulative temperature or sine function [5],

$w=\hat{w}\tau^{r}\left[ 1-\left[ \max\left( 0, 1-\left( 1-q \right)\frac{G\left( t \right)-G(t_{0})}{\tau} \right) \right]^{\frac{1}{1-q}} \right]^{r}$, (A)

$G\left( t \right)=t+\frac{\alpha}{2\pi}\sin2\pi\left( t-t_{1} \right)$, (B)

where *α* (*α* ≥ 0) and *t*_1_ (0 ≤ *t*_1_ ≤ 1) manipulate the amplitude and phase of seasonal variation, respectively. An example of the growth trajectory by equations A and B with seasonal variation in growth rates is shown in S2 Figure.

In addition, we can set some constraints for the generalized *q*-VBGF to pass through certain points (e.g. size at birth, size at maturity, maximum size) to increase the robustness of estimation as follows. By setting *w* = *w*_1_ at a certain age *t* = *t*_1_ in a life history, equation 12 can be deformed to the following equation:

$w=w_{1}\left[ \frac{1-\left[ \max\left( 0,1-(1-q)\frac{t-t_{0}}{\tau} \right) \right]^{\frac{1}{1-q}}}{1-\left[ \max\left( 0,1-(1-q)\frac{t_{1}-t_{0}}{\tau} \right) \right]^{\frac{1}{1-q}}} \right]^{r}$. (C)

When *t*_1_ is the age at maturity, then *w*_1_ is the body size at maturity, which allows the growth function to include a practical life history parameter instead of *w_∞_* or *ŵ*. Note that at least one of *t*_1_ or *w*_1_ is not a parameter to be estimated; rather, it must be set beforehand from external information.

If we set the coordinate to pass through (*t*_1_, *w*_1_) instead of the origin (*t*_1_, 0), equation B can be deformed as follows (see Appendix C for derivation):

$w=\hat{w}\tau^{r}\left[ 1-\left[ \max\left( 0,\left[ 1-\frac{1}{\tau}\left( \frac{w_{1}}{\hat{w}} \right)^{\frac{1}{r}} \right]^{1-q}-\left( 1-q \right)\frac{t-t_{1}}{\tau} \right) \right]^{\frac{1}{1-q}} \right]^{r}$. (D)

It will be useful if we set (*t*_1_, *w*_1_) as the age and body size at birth.

By using a similar method to the one described by Schnute [48], i.e., setting two certain coordinates in a life history, (*t*_1_, *w*_1_) and (*t*_2_, *w*_2_), where *t*_1_ < *t*_2_, for the growth curve to pass through, we can convert equation 12 as follows (see Appendix D for derivation):

$w=\left[ {w_{1}}^{\frac{1}{r}}+\left( {w_{2}}^{\frac{1}{r}}-{w_{1}}^{\frac{1}{r}} \right)\frac{1-\left[ \max\left( 0,1-(1-q)\frac{t-t_{1}}{T} \right) \right]^{\frac{1}{1-q}}}{1-\left[ \max\left( 0,1-(1-q)\frac{t_{2}-t_{1}}{T} \right) \right]^{\frac{1}{1-q}}} \right]^{r}$. (E)

Note that the maturation timing parameter *T* is different from the original parameter *τ*. The value of *τ* can be calculated by the estimated parameter values from equation E as follows (see Appendix D for derivation):

$\tau=\left[ \frac{{w_{2}}^{\frac{1}{r}}T^{\frac{1}{1-q}}-{w_{1}}^{\frac{1}{r}}\left[ \max\left( 0, T-\left( 1-q \right)\left( t_{2}-t_{1} \right) \right) \right]^{\frac{1}{1-q}}}{{w_{2}}^{\frac{1}{r}}-{w_{1}}^{\frac{1}{r}}} \right]^{1-q}$. (F)

**Appendix A: Derivation of equation 9**

Replacing the standardized body size (*w*/*w*_∞_)^1/r^ in equation 6 into *X* derives

$\frac{dX}{dt}=\frac{k}{r{w_{\infty}}^{\frac{1}{r}}}\left( 1-X \right)^{q}$,

$\int\left( 1-X \right)^{-q}dX=\int\frac{k}{r{w_{\infty}}^{\frac{1}{r}}}dt$.

Given that *X* → 0 when *t* → *t*_0_, then

$\frac{\left( 1-X \right)^{1-q}-1}{q-1}=\frac{k}{r{w_{\infty}}^{\frac{1}{r}}}(t-t_{0})$,

$X=1-\left[ 1-\left( 1-q \right)\frac{k}{r{w_{\infty}}^{\frac{1}{r}}}\left( t-t_{0} \right) \right]^{\frac{1}{1-q}}$.

Thus,

$w=w_{\infty}\left[ 1-\left[ 1-\frac{k}{r{w_{\infty}}^{\frac{1}{r}}}\left( 1-q \right)\left( t-t_{0} \right) \right]^{\frac{1}{1-q}} \right]^{r}$. (28)

**Appendix B: Extension of equations 18 and 19 into the generalized *q*-VBGF in equation 17**

Replacing the first and second exponentials in equation 18, *w*=*w*_∞_exp[−exp[−*K*(*t*−*t*_0_)]], into *q*-exponentials exp_(1-1/_*_r_*_)_ and exp*_q_* derives

$w=w_{\infty}\exp_{1-\frac{1}{r}} \left[ -\exp_{q} \left[ -K\left( t-t_{0} \right) \right] \right]$. (G)

From the definition of *q*-exponential

$\exp_{q} x:=\left[ 1+\left( 1-q \right)x \right]^{\frac{1}{1-q}}$, (16)

Equation G can be reformed as:

$w=w_{\infty}\exp_{1-\frac{1}{r}}\left[ -\left[ 1-\left( 1-q \right)K(t-t_{0}) \right]^{\frac{1}{1-q}} \right]$.

By re-parameterizing $K\to\frac{r^{1-q}}{\tau}$ and $t_{0}\to t_{0}-\frac{\tau}{r^{1-q}}\frac{1-r^{1-q}}{1-q}$:

$w=w_{\infty}\exp_{1-\frac{1}{r}}\left[ -\left[ 1-\left( 1-q \right)\frac{r^{1-q}}{\tau}\left( t-t_{0}+\frac{\tau}{r^{1-q}}\frac{1-r^{1-q}}{1-q} \right) \right]^{\frac{1}{1-q}} \right]$,

$w=w_{\infty}\exp_{1-\frac{1}{r}}\left[ -\left[ r^{1-q}-\left( 1-q \right)\frac{r^{1-q}}{\tau}\left( t-t_{0} \right) \right]^{\frac{1}{1-q}} \right]$,

$w=w_{\infty}\exp_{1-\frac{1}{r}}\left[ -{r\left[ 1-\left( 1-q \right)\frac{\left( t-t_{0} \right)}{\tau} \right]}^{\frac{1}{1-q}} \right]$,

$w=w_{\infty}\exp_{1-\frac{1}{r}}\left[ -r\exp_{q} \left[ -\frac{t-t_{0}}{\tau} \right] \right]$. (17)

Thus, equation 17 is derived.

Similarly, equation 19, *w* = *w*_∞_ [1 + *p* exp[−*K*(*t*−*t*_0_)]] ^−1/^*^p^*, can be reformed as:

$w=w_{\infty}\exp_{1+p} \left[ -\exp\left[ -K\left( t-t_{0} \right) \right] \right]$.

Replacing the exponential in the bracket into the *q*-exponential exp*_q_* derives

$w=w_{\infty}\exp_{1+p} \left[ -\exp_{q} \left[ -K\left( t-t_{0} \right) \right] \right]$. (H)

By re-parameterizing $K\to\frac{r^{1-q}}{\tau}$, $t_{0}\to t_{0}-\frac{\tau}{r^{1-q}}\frac{1-r^{1-q}}{1-q}$, and *p* → −1/*r*, and re-arranging the form:

$w=w_{\infty}\exp_{1-\frac{1}{r}}\left[ -r \exp_{q}\left[ -\frac{t-t_{0}}{\tau} \right] \right]$. (17)

Thus, equation 17 is derived.

**Appendix C: Derivation of equation D**

Equation 12 can be deformed as

$w=\hat{w}\tau^{r}\left[ 1-\left[ \max\left( 0, 1-\left( 1-q \right)\frac{t-t_{1}}{\tau}-\left( 1-q \right)\frac{t_{1}-t_{0}}{\tau} \right) \right]^{\frac{1}{1-q}} \right]^{r}$. (I)

If equation I passes through (*t*_1_, *w*_1_), then,

$w_{1}=\hat{w}\tau^{r}\left[ 1-\left[ \max\left( 0, 1-\left( 1-q \right)\frac{t_{1}-t_{0}}{\tau} \right) \right]^{\frac{1}{1-q}} \right]^{r}$.

If *t*_1_ is small enough to satisfy 1− (1−*q*)(*t*_1_−*t*_0_)/*τ* ≥ 0, then,

$w_{1}=\hat{w}\tau^{r}\left[ 1-\left[ 1-\left( 1-q \right)\frac{t_{1}-t_{0}}{\tau} \right]^{\frac{1}{1-q}} \right]^{r}$,

$1-\left( 1-q \right)\frac{t_{1}-t_{0}}{\tau}=\left[ 1-\frac{1}{\tau}\left( \frac{w_{1}}{\hat{w}} \right)^{\frac{1}{r}} \right]^{1-q}$. (J)

Substituting equation J into I derives

$w=\hat{w}\tau^{r}\left[ 1-\left[ \max\left( 0, \left[ 1-\frac{1}{\tau}\left( \frac{w_{1}}{\hat{w}} \right)^{\frac{1}{r}} \right]^{1-q}-\left( 1-q \right)\frac{t-t_{1}}{\tau} \right) \right]^{\frac{1}{1-q}} \right]^{r}$. (D)

Thus, equation D is derived.

**Appendix D: Derivation of equations E and F**

If *t*_1_ is small enough to satisfy 1− (1−*q*)(*t*_1_−*t*_0_)/*τ* ≥ 0, equation I can be converted as:

$w^{\frac{1}{r}}=\hat{w}^{\frac{1}{r}}\tau\left[ 1-\left[ 1-\left( 1-q \right)\frac{t_{1}-t_{0}}{\tau} \right]^{\frac{1}{1-q}}\left[ \max\left( 0, \frac{1-\left( 1-q \right)\frac{t_{1}-t_{0}}{\tau}}{1-\left( 1-q \right)\frac{t_{1}-t_{0}}{\tau}}-\frac{\left( 1-q \right)\frac{t-t_{1}}{\tau}}{1-\left( 1-q \right)\frac{t_{1}-t_{0}}{\tau}} \right) \right]^{\frac{1}{1-q}} \right]$,

$w^{\frac{1}{r}}=\hat{w}^{\frac{1}{r}}\tau\left[ 1-\left[ 1-\left( 1-q \right)\frac{t_{1}-t_{0}}{\tau} \right]^{\frac{1}{1-q}}\left[ \max\left( 0, 1-\left( 1-q \right)\frac{t-t_{1}}{\tau-\left( 1-q \right)\left( t_{1}-t_{0} \right)} \right) \right]^{\frac{1}{1-q}} \right]$.

By substituting equation J,

$w^{\frac{1}{r}}=\hat{w}^{\frac{1}{r}}\tau\left[ 1-\left[ 1-\frac{1}{\tau}\left( \frac{w_{1}}{\hat{w}} \right)^{\frac{1}{r}} \right]\left[ \max\left( 0,1-\left( 1-q \right)\frac{t-t_{1}}{\tau-\left( 1-q \right)\left( t_{1}-t_{0} \right)} \right) \right]^{\frac{1}{1-q}} \right]$,

$w^{\frac{1}{r}}=\hat{w}^{\frac{1}{r}}\tau-\left( \hat{w}^{\frac{1}{r}}\tau-{w_{1}}^{\frac{1}{r}} \right)\left[ \max\left( 0, 1-\left( 1-q \right)\frac{t-t_{1}}{\tau-\left( 1-q \right)\left( t_{1}-t_{0} \right)} \right) \right]^{\frac{1}{1-q}}$,

$w^{\frac{1}{r}}-{w_{1}}^{\frac{1}{r}}=\left( \hat{w}^{\frac{1}{r}}\tau-{w_{1}}^{\frac{1}{r}} \right)\left[ {1-\left[ \max\left( 0, 1-\left( 1-q \right)\frac{t-t_{1}}{\tau-\left( 1-q \right)\left( t_{1}-t_{0} \right)} \right) \right]}^{\frac{1}{1-q}} \right]$. (K)

If equation K passes through (*t*_2_, *w*_2_), then,

${w_{2}}^{\frac{1}{r}}-{w_{1}}^{\frac{1}{r}}=\left( \hat{w}^{\frac{1}{r}}\tau-{w_{1}}^{\frac{1}{r}} \right)\left[ {1-\left[ \max\left( 0, 1-\left( 1-q \right)\frac{t_{2}-t_{1}}{\tau-\left( 1-q \right)\left( t_{1}-t_{0} \right)} \right) \right]}^{\frac{1}{1-q}} \right]$. (L)

Dividing equation K by L derives

$w^{\frac{1}{r}}-{w_{1}}^{\frac{1}{r}}=\left( {w_{2}}^{\frac{1}{r}}-{w_{1}}^{\frac{1}{r}} \right)\frac{{1-\left[ \max\left( 0, 1-\left( 1-q \right)\frac{t-t_{1}}{\tau-\left( 1-q \right)\left( t_{1}-t_{0} \right)} \right) \right]}^{\frac{1}{1-q}}}{{1-\left[ \max\left( 0, 1-\left( 1-q \right)\frac{t_{2}-t_{1}}{\tau-\left( 1-q \right)\left( t_{1}-t_{0} \right)} \right) \right]}^{\frac{1}{1-q}}}$. (M)

By re-parameterizing *τ* − (1-*q*) (*t*_1_-*t*_0_) → *T*, we get

$w=\left[ {w_{1}}^{\frac{1}{r}}+\left( {w_{2}}^{\frac{1}{r}}-{w_{1}}^{\frac{1}{r}} \right)\frac{1-\left[ \max\left( 0,1-(1-q)\frac{t-t_{1}}{T} \right) \right]^{\frac{1}{1-q}}}{1-\left[ \max\left( 0,1-(1-q)\frac{t_{2}-t_{1}}{T} \right) \right]^{\frac{1}{1-q}}} \right]^{r}$. (E)

Thus, equation E is derived.

From T = *τ* − (1-*q*)(*t*_1_-*t*_0_),

$t_{0}=t_{1}+\frac{T-\tau}{1-q}$. (N)

By substituting (*t*, *w*) = (*t*_2_, *w*_2_) into equation C,

$w_{2}=w_{1}\left[ \frac{1-\left[ \max\left( 0,1-(1-q)\frac{t_{2}-t_{0}}{\tau} \right) \right]^{\frac{1}{1-q}}}{1-\left[ \max\left( 0,1-(1-q)\frac{t_{1}-t_{0}}{\tau} \right) \right]^{\frac{1}{1-q}}} \right]^{r}$. (O)

Substituting equation N into O derives

$w_{2}=w_{1}\left[ \frac{1-\left[ \max\left( 0,\frac{T}{\tau}-(1-q)\frac{t_{2}-t_{1}}{\tau} \right) \right]^{\frac{1}{1-q}}}{1-\left[ \max\left( 0,\frac{T}{\tau} \right) \right]^{\frac{1}{1-q}}} \right]^{r}$,

$w_{2}=w_{1}\left[ \frac{\tau^{\frac{1}{1-q}}-\left[ \max\left( 0,T-(1-q)\left( t_{2}-t_{1} \right) \right) \right]^{\frac{1}{1-q}}}{\tau^{\frac{1}{1-q}}-T^{\frac{1}{1-q}}} \right]^{r}$,

$\left( \frac{w_{2}}{w_{1}} \right)^{\frac{1}{r}}\left( \tau^{\frac{1}{1-q}}-T^{\frac{1}{1-q}} \right)=\tau^{\frac{1}{1-q}}-\left[ \max\left( 0, T-\left( 1-q \right)\left( t_{2}-t_{1} \right) \right) \right]^{\frac{1}{1-q}}$,

$\tau^{\frac{1}{1-q}}\left[ \left( \frac{w_{2}}{w_{1}} \right)^{\frac{1}{r}}-1 \right]=\left( \frac{w_{2}}{w_{1}} \right)^{\frac{1}{r}}T^{\frac{1}{1-q}}-\left[ \max\left( 0, T-\left( 1-q \right)\left( t_{2}-t_{1} \right) \right) \right]^{\frac{1}{1-q}}$,

$\tau=\left[ \frac{\left( \frac{w_{2}}{w_{1}} \right)^{\frac{1}{r}}T^{\frac{1}{1-q}}-\left[ \max\left( 0, T-\left( 1-q \right)\left( t_{2}-t_{1} \right) \right) \right]^{\frac{1}{1-q}}}{\left( \frac{w_{2}}{w_{1}} \right)^{\frac{1}{r}}-1} \right]^{1-q}$,

$\tau=\left[ \frac{{w_{2}}^{\frac{1}{r}}T^{\frac{1}{1-q}}-{w_{1}}^{\frac{1}{r}}\left[ \max\left( 0, T-\left( 1-q \right)\left( t_{2}-t_{1} \right) \right) \right]^{\frac{1}{1-q}}}{{w_{2}}^{\frac{1}{r}}-{w_{1}}^{\frac{1}{r}}} \right]^{1-q}$. (F)

Thus, equation F is derived.

**References**

1. Kato H. Density dependent changes in growth parameters of the southern minke whale. Sci. Rep. Whales Res. Inst. 1987; 38: 47-7.
2. Ueda Y, Yomatsu I, Fujiwara K, Matsukura R, Yamada T, Yamamoto T. Stock assessment of the snow crab subpopulation in the Sea of Japan in 2015 FY. In: Assessment of fisheries stocks in the waters around Japan in 2015 FY Part. Tokyo: Fisheries Agency of Japan and Japan Fisheries Research and Education Agency; 2015. Pp 545-610. (in Japanese).
3. Yamamoto T, Yamada T, Kinoshita T, Ueda Y, Fujimoto H, Yamasaki A, Hamasaki K. Effects of temperature on growth of juvenile snow crabs, *Chionoecetes opilio*, in the laboratory. J. Crustacean Biol. 2015; 35: 140-148.
4. Schnute J. A versatile growth model with statistically stable parameters. Can. J. Fish. Aquat. Sci. 1981; 38: 1128-1140. (doi:10.1139/f81-153)
